# Common themes in antimicrobial and anticancer drug resistance

**DOI:** 10.3389/fmicb.2022.960693

**Published:** 2022-08-08

**Authors:** Mariana Carmen Chifiriuc, Roxana Filip, Marian Constantin, Gratiela Gradisteanu Pircalabioru, Coralia Bleotu, Liliana Burlibasa, Elena Ionica, Nicolae Corcionivoschi, Grigore Mihaescu

**Affiliations:** ^1^Faculty of Biology, University of Bucharest, Bucharest, Romania; ^2^Life, Environmental and Earth Sciences Division, Research Institute of the University of Bucharest, Bucharest, Romania; ^3^The Romanian Academy, Bucharest, Romania; ^4^Academy of Romanian Scientists, Bucharest, Romania; ^5^Faculty of Medicine and Biological Sciences, Stefan cel Mare University of Suceava, Suceava, Romania; ^6^Suceava Emergency County Hospital, Suceava, Romania; ^7^Institute of Biology, Bucharest, Romania; ^8^Stefan S. Nicolau Institute of Virology, Bucharest, Romania; ^9^Romanian Academy of Scientists, Bucharest, Romania; ^10^Bacteriology Branch, Veterinary Sciences Division, Agri-Food and Biosciences Institute, Belfast, United Kingdom; ^11^Faculty of Bioengineering of Animal Resources, Banat University of Agricultural Sciences and Veterinary Medicine—King Michael I of Romania, Timisoara, Romania

**Keywords:** microbial biofilms, persister cells, efflux pumps, stress response, gut microbiota, mutator phenotype

## Abstract

Antimicrobial and anticancer drug resistance represent two of the main global challenges for the public health, requiring immediate practical solutions. In line with this, we need a better understanding of the origins of drug resistance in prokaryotic and eukaryotic cells and the evolutionary processes leading to the occurrence of adaptive phenotypes in response to the selective pressure of therapeutic agents. The purpose of this paper is to present some of the analogies between the antimicrobial and anticancer drug resistance. Antimicrobial and anticancer drugs share common targets and mechanisms of action as well as similar mechanisms of resistance (e.g., increased drug efflux, drug inactivation, target alteration, persister cells’ selection, protection of bacterial communities/malignant tissue by an extracellular matrix, etc.). Both individual and collective stress responses triggered by the chemotherapeutic agent involving complex intercellular communication processes, as well as with the surrounding microenvironment, will be considered. The common themes in antimicrobial and anticancer drug resistance recommend the utility of bacterial experimental models for unraveling the mechanisms that facilitate the evolution and adaptation of malignant cells to antineoplastic drugs.

## Introduction

The emerging and accelerated evolution of drug resistance in microbial and tumor cells share many analogies such as common targets (topoisomerase II) and mechanisms of action; availability of molecules with dual antimicrobial and antineoplastic activity (e.g., antineoplastic antibiotics); similar mechanisms of resistance; common selective drivers for resistance; dual resistance, respectively, intrinsic (failure to obtain an initial response to drugs) and acquired (involving specific and gradual genetic and epigenetic mechanisms); and existence of complex interactions between the gut microbiota and the drugs (Lambert et al., 2011). We will enumerate below some of the most well-known examples of similarities between antimicrobial and anticancer drugs resistance, at single cell and population level, that will be further detailed in dedicated sections of this review.

### Single-cell processes involved in antimicrobial and anticancer drugs resistance

One of the best-known examples of multi-drug resistance (MDR) mechanisms shared by bacterial and malignant cells are the efflux pumps (Spratt, 1994; Davies and Davies, 2010). Efflux pump activation is responsible for therapeutic failure in solid and hematological malignancies as well as in many bacterial infections (Sissi and Palumbo, 2003; Bisacchi and Hale, 2016). Another common feature is represented by the adaptation strategies to the stress induced by different microenvironments, which are triggered by the genetic instability of tumor cells and bacterial mutability, respectively. In this regard, stimulation of stress-induced DNA repairing (SOS) genes plays an important role in DNA repair in both prokaryotic and eukaryotic cells exposed to inhibitory agents. Bacterial resistance could occur not only by spontaneous mutations or horizontal gene transfer of resistance genes but also by adaptation through prolonged exposure to sublethal doses (Andersson and Hughes, 2014). It is very likely that the same adaptive mechanism is at work in neoplastic cells.

### Collective responses involved in antimicrobial and anticancer drugs resistance

Besides single-cell processes, adaptation at population level has been shown to be involved in resistance to both antimicrobial and antineoplastic agents. The collective stress response triggered by the chemotherapeutic agent involves complex intercellular communication processes, as well as with the surrounding microenvironment (Stewart and Costerton, 2001; Butler et al., 2010). For example, in the absence of nutrients, both microbial and tumor cells adapt to environmental conditions by metabolically shifting to a persistent phenotype in which cells do not grow and divide. In the presence of antibiotics, bacterial cells try to avoid the drugs by flagellar movement to a region of low concentration of the cytocidal agent and by forming a biofilm, which limits access of the drug (Stewart and Costerton, 2001; Butler et al., 2010). The tumor cells avoid the antineoplastic drugs by metastasis or by establishing an altered microenvironment through vascularization (Ahmed et al., 2010; Lambert et al., 2011). The stroma and extracellular matrix (ECM) of neoplastic cells and the microbial biofilm exopolymeric matrix limit the rate of O_2_ and nutrient diffusion and protect the cellular communities.

### Interaction of antimicrobial and anticancer drugs with gut microbiota

The gut microbiome plays an important role in modulating the efficacy and toxicity of antibiotics and antitumoral agents, thus representing an attractive target for improving drug safety and efficacy, by manipulating its composition. However, both antibacterial and anticancer treatments induce perturbations in the normal microbiota (also known as dysbiosis). Dysbiosis is not only associated with diarrhea and fungal infections, it could have a role in neoplastic etiology and cancer risk, influence the efficacy of chemotherapy, radiotherapy, and immunotherapy, and also promote the emergence of antimicrobial and antitumoral drug resistance (Alexander et al., 2017; Lazar et al., 2019; Pinato et al., 2019; Cheng et al., 2020). Antibiotics with broad spectrum have been shown to induce numerical changes in 3% of bacterial species in the gut microbiota and the occurred dysbiosis further alters the therapeutic efficacy of certain antitumor antibiotics and increases their toxicity (Lofmark et al., 2006; Jernberg et al., 2007; Andersson and Hughes, 2014; Francino, 2015). On the other side, antineoplastic chemotherapy indirectly amplifies dysbiosis and has profound effects, mainly on the intestinal epithelium, influencing cancer progression, treatment efficacy, and toxicity.

All these functional analogies and complex interactions can generate common therapeutic strategies. Therefore, the purpose of this paper is to present some of the analogies between the antimicrobial and anticancer drug resistance, starting with the presentation of the common targets and mechanisms of action and of the antineoplastic antibiotics. Both single-cell and collective mechanisms of resistance in bacterial communities and malignant tissues will be presented.

## Antimicrobial and anticancer drug resistance: The dimension of the challenge for the public health

Nowadays, cancer and infectious diseases are two of the most problematic and common diseases, exhibiting a great impact on the health status of the general population. According to the World Health Organization (WHO), cancer is one of the leading mortality causes worldwide, with nearly 10 million deaths recorded in 2020.^1^ The most common neoplasia, also accounting for the highest mortality rates are, in alphabetical order, breast, colorectal, liver, lung, prostate, skin, and stomach cancers (Ferlay et al., 2021). The current therapeutic approaches in cancer include surgery and chemotherapy as first options, followed by radiotherapy, immunotherapy, and targeted therapy (Kanady et al., 1997). Based on experimental tumor induction and propagation models, it is estimated that in neoplasms containing 10^3^–10^6^ cells, the chemotherapeutic treatment can select at least one clone of resistant cells, which can survive and continue to proliferate, leading to tumor recurrence or metastasis formation. The spontaneous mutation rate is directly proportional with the metastatic potential, being 3- to 7-fold higher in case of tumorigenic clones with high metastatic potential (Cifone and Fidler, 1981). Thus, the emergence of resistance to antitumoral drugs currently represents one of the major medical challenges.

Antimicrobial resistance (AMR) is one of the top ten global threats, not only for humans, but also for the environmental health, being considered a typical One Health problem.^2^ The most threatening bugs, exhibiting MDR, extended-drug and even pan-drug resistance phenotypes are grouped under the acronym ESCAPE (*Enterococcus faecium*, *Staphylococcus aureus, Clostridium difficile, Acinetobacter baumannii, Pseudomonas aeruginosa*, and *Enterobacteriaceae*; Peterson, 2009). The first 18 most fearful resistant microbes are classified by the Center for Disease Control and Prevention (CDC) as either urgent, serious, or concerning threats in the 2019 AR Threats Report (CDC’s Antibiotic Resistance Threats in the United States, 2019).

The antimicrobials crisis, beyond direct consequences related to increased mortality and morbidity rates caused by infectious diseases and huge economic losses, would compromise the success of modern medicine, including invasive diagnosis procedures (e.g., biopsy), surgery, and cancer radio- and chemotherapy. Regardless of the extent of the surgery, the risk of developing healthcare associated infections with resistant strains is very high, causing difficulties in making the most appropriate therapeutic decision. Also, radiotherapy and chemotherapy could induce a temporary immunodeficiency and thus increase the oncologic patient susceptibility to infections, including those produced by MDR bacteria^3^ (Ariza-Heredia and Chemaly, 2018). Fluoroquinolone and carbapenem resistance, nosocomial outbreaks of Gram-negative sepsis on cancer wards, are often caused by enteric bacteria, with mortality rates of 60%–84% (Papanicolas et al., 2018).

All these facts demonstrate that the continuously growing cancer burden is potentiated by the emergence and spread of AMR.

## Common features in antimicrobial and antineoplastic agents’ structures, targets, and mechanisms of action

**Figure 1:**
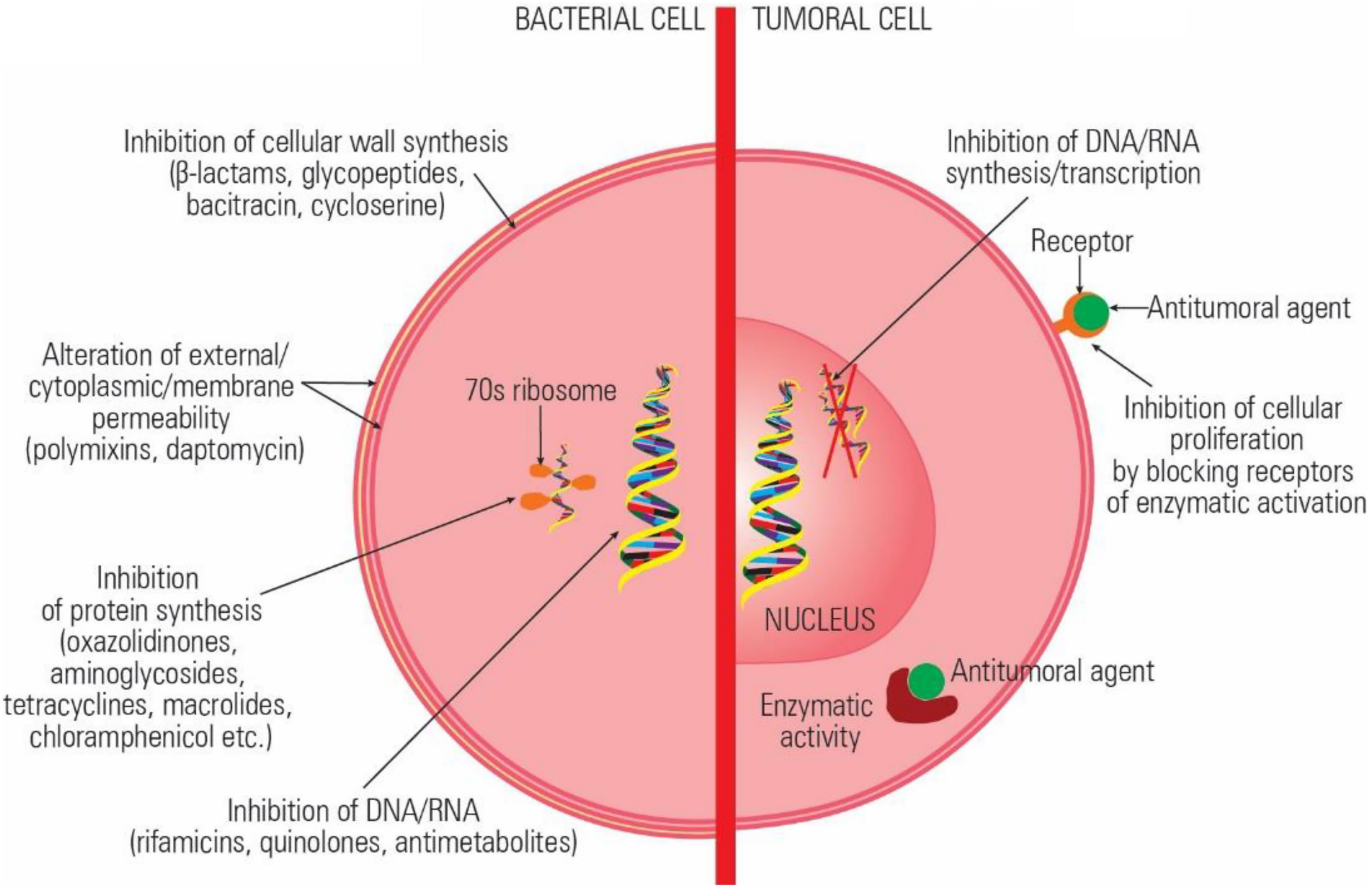
Comparative representation of the antibiotics and antitumoral agents’ mechanisms of action.

The currently available antibiotics act through: (a) inhibition of cell wall synthesis (beta-lactams); (b) alteration of cell surface structures (cell wall, outer membrane, cytoplasmic membrane; polypeptides, daptomycin), (c) inhibition of protein synthesis (aminoglycosides, tetracyclines, macrolides, lincosamides, streptogramins, oxazolidinones, chloramphenicol, etc.) (d) inhibition of DNA synthesis and transcription (quinolones, rifamycins); and inhibition of essential metabolites (trimethoprim-sulfamethoxazole; Mihaescu et al., 2008, 2009). The main mechanisms of action of the antitumoral drugs are (a) alteration of DNA structure by intercalation or ROS release, (b) inhibition of enzymes involved in replication and transcription, (c) inhibition of essential metabolites, and (d) inhibition of cellular growth by preventing the activation of certain proteins (Figure 1).

Some of the current antibiotics were reported to have the same target and similar mechanisms of action exhibiting both antimicrobial and antitumor activity (Gao et al., 2020). Antineoplastic antibiotics with major therapeutic impact are anthracyclines, peptides, and quinolones, which interact with topoisomerases both in prokaryotic and eukaryotic cells (Pommier et al., 2010). Anthracyclines react with topoisomerase DNA complex, stabilize it, and induce cell apoptosis (D’Arpa and Liu, 1989). Quinolones and aminocoumarin antibiotics target type II topoisomerases (gyrase) and topoisomerase IV, converting these enzymes into physiological poisons, both in tumoral and bacterial cells (Barrett, 1996; Salerno et al., 2010). Moreover, besides their direct antitumoral effects, quinolones proved to have immunomodulators activity and to stimulate the antineoplastic immune response (Dalhoff and Shalit, 2003).

These aspects suggest that the identification of inhibitory compounds with multiple targets (i.e., topoisomerases I and II and DNA repairing enzymes) could represent a promising lead to efficiently fight bacterial infections and cancer and to avoid emergence of bacterial and tumor cells resistance (Skok et al., 2020). Even a low level of inhibition for multiple bacterial and tumor cell functions would have a high cumulative effect and limit the risk of resistance development (Avner et al., 2012).

## Common features in antimicrobial and antineoplastic agents’ resistance

**Figure 2:**
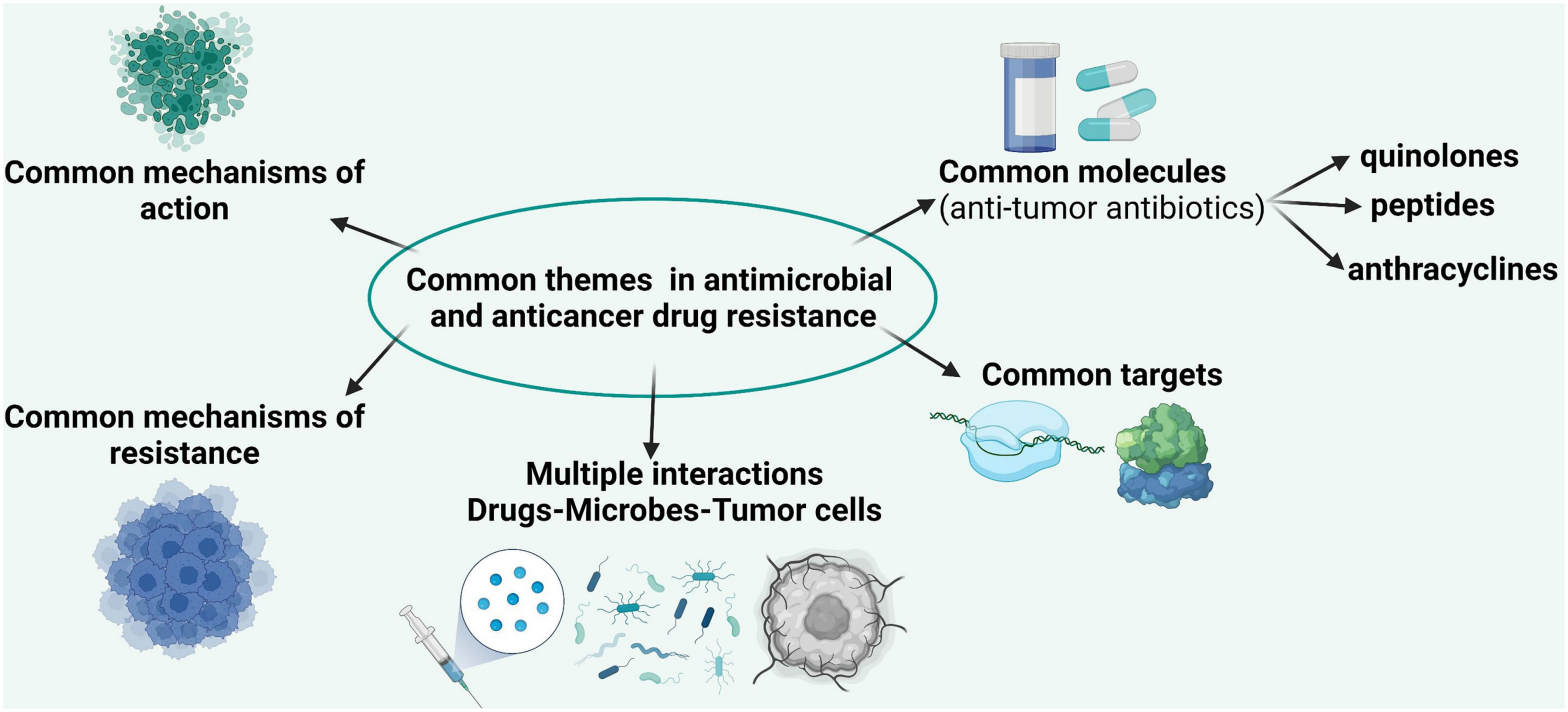
Comparative representation of the main targets, mechanisms of action, and mechanisms of resistance in bacterial and tumoral cells.

The accelerated evolution of resistance of neoplastic cells to chemotherapeutic agents and of bacterial cells to antibiotics is the result of common mechanisms acting at individual (Figure 2) and population levels (Lambert et al., 2011). The current research is focused on the identification of these mechanisms in order to be able to develop efficient drugs to overcome them (Nikolaou et al., 2018).

### Single-cell resistance mechanisms

After surgical resection of the primary tumor, the next step in the therapeutic approach is chemotherapy. This can be given before and/or after the primary tumor is resected to inhibit the proliferation of malignant cells and induce their apoptosis, but its disadvantage is the selection of resistant clones. According to Goldie–Coldman, the clones of tumor resistant cells survive because of spontaneous mutations, which occur in 1/1,000,000 cells; thus, the probability of the emergence of tumor resistance is very high (considering that 1 g of tumor tissue contains 10^9^ cells) (Goldie and Coldman, 1979, 1983, 1984). In bacteria, resistance is acquired either by spontaneous mutations or by horizontal gene transfer mediated by phage transduction, transformation with external DNA, and resistance plasmid transfer during conjugation.

Malignant cells could develop resistance through one/some of the following biochemical mechanisms: (a) inhibition of drug distribution through circulation or blood–brain barrier, (b) inhibition of intracellular accumulation, (c) inhibition of interaction between the chemotherapeutic agent and target molecules (i.e., proteins and nucleic acids), (d) transformation of the active agent or of its specific target, (e) enzymatic or chemical drug inactivation, and (f) removal of the chemotherapeutic agent from the cells *via* efflux pumps.

In bacteria, the biochemical mechanisms of resistance include (a) decreased porin permeability, (b) active efflux of the antibiotic, (c) target alteration, and (d) chemical modification or inactivation of antibiotics (Patel et al., 2021). Thus, one of the most important resistance mechanisms present in both bacteria and tumor cells is represented by the activation/hyperactivation of efflux pumps. In neoplastic cells, the overexpression of P-glycoprotein occurred through the amplification of the encoding gene is leading to MDR to hydrophobic compounds: (a) anthracyclines (doxorubicin, daunorubicin, epirubicin, and idarubicin); (b) aminoacridines (AMSA); (c) taxans (taxol, etc.), vinca alkaloids, and actinomycin D; and (d) mitomycin C and topoisomerase I inhibitors (Patel et al., 2021) In bacteria, there are at least four families of secondary transporters conferring MDR and assuring toxic compounds elimination (Mihaescu et al., 2008).

### Bacterial biofilms versus tumoral tissue – The power of the collective

**Figure 3:**
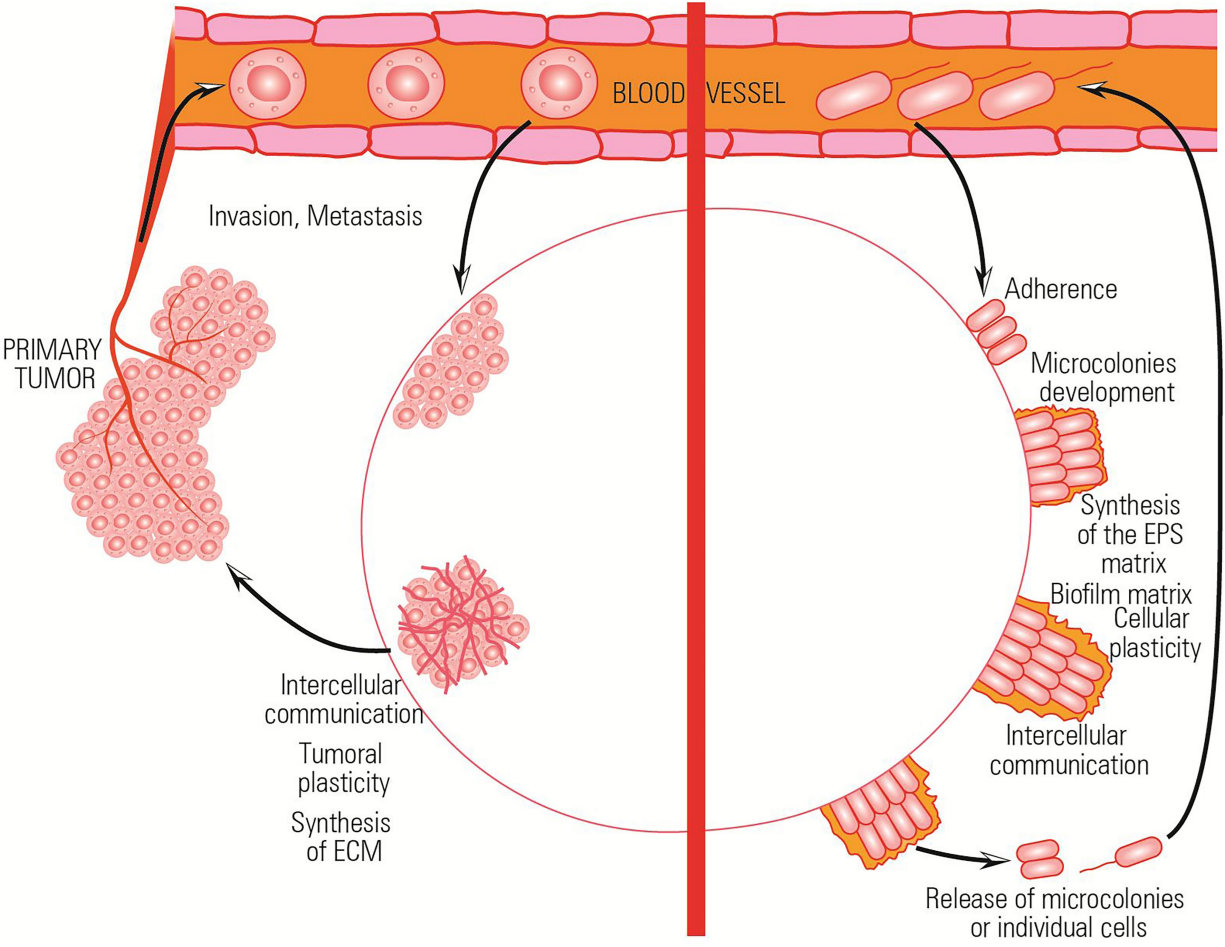
Comparative representation of the collective adaptative mechanisms exhibited by bacterial and tumor cells in stressful environments.

The ability to resist a chemotherapeutic treatment or to survive in stressful environments is the result of the ability of both bacterial and tumor cells to cooperate and elaborate a collective adaptive response (Figure 3).

Both bacterial and tumor cells adapt to different microenvironmental conditions by changing their phenotype and activating the stress response processes. For example, in the absence of nutrients, both bacterial and tumor cells adapt to environmental conditions by metabolically shifting to a persister phenotype in which cells do not grow and divide.

Also, both bacterial and tumor cells transiently increase their mutation rates (e.g., hypermutator bacterial cells) and can reduce DNA replication fidelity to foster diversity. This process is known as adaptive mutability and leads to accelerated evolution and adaptation. As a result, biofilm and tumor cells could develop MDR much more rapidly. However, the hypermutability state is maintained only in a small population fraction reducing the risk of detrimental or fatal mutations in the rest of the clonal population (Taddei et al., 1997; Macia et al., 2005).

Both bacterial and tumor cells adopt a running behavior (mediated by flagellar motility in case of bacteria and by metastasis process in case of tumoral cells) in the presence of stressor agents.

The bacterial communities/tumoral tissues isolate in citadels which are hard to penetrate by the antibiotics/antitumoral agents. Inside these isolated communities, a collective stress response, involving complex intercellular and with the surrounding microenvironment communication, is achieved (Lambert et al., 2011). All these functional analogies and a better understanding of the mechanisms and implications of individual and collective adaptive evolution are of relevance to generate common therapeutic strategies (Table 1).

**Table 1:** Summarization of comparative mechanisms adopted by bacterial and tumor cells seeking survival under stress (Lambert et al., 2011).

Mechanisms to escape stress mediated by drug exposure	Bacteria	Tumors
Phenotypic, reversible mechanisms		
Moving to an environment that contains a lower concentration of a cytocidal agent	Mobility	Metastasis
The cell population can create a milieu where the drug has limited access to the cells	Biofilms	Altered tumor microenvironment (including the vasculature)
	The extracellular polymeric matrix limits the influx of nutrients and oxygen	Tumor stroma and dense surrounding extracellular matrix limiting the diffusion of nutrients and oxygen
	Intercellular communication inside biofilm cells and with the environment through quorum sensing and response	Intercellular communication inside tumor cells and with different cells from the surrounding healthy tissues through direct contact and soluble mediators (cytokines)
	Biofilm compartmentalization as response to different phsyico-chemical conditions leading to phenotypic heterogeneity/primitive differentiation	Highly specialized cells and heterogeneous population Metabolic shift toward anaerobic glycolysis
	Lysis of bacterial cells and occurrence of a channel network/cavities facilitating the water and nutrients access to the inner biofilm layers	Tumor neovascularization
	Isolated cells/microcolonies dispersal and initiation of a novel biofilm in other anatomic sites	Metastatic expansion from a primary human tumor
Phenotypic switching	Persisters’ phenotype	
Genetic variation (inherent/*de novo* mutations)		
Stress-induced mutagenesis (activation of SOS response)	Selection of (hyper) mutator (high mutation rates) phenotypes Genetic instability in cancer tissues assuring rapid and high adaptability to microenvironment changes and drugs	

## Gut microbiota–antibiotics–antitumoral agents trialogue

Given the similarities regarding the antibiotics and some antitumoral agents’ structure, targets, and resistance mechanisms, concerns have been raised regarding their bidirectional interactions with the host microbiota. The human microbiota is considered both an essential organ, with important roles in nutrition, pathogenesis, and immunity, and the second genome, represented by bacteria, archaea, fungi, and viruses (Neu, 2015).

The normal gut microbiota has a density of 10^11^–10^12^ cells/ml content and contains mainly two anaerobic phylotypes: *Bacteroidetes* (48%) and *Firmicutes* (51%) together with *Proteobacteria, Fusobacteria, Spirochaetes,* etc. (Mandal et al., 2016). The intestinal microbiome plays an important role in integration of the dietary signals with immune system reactivity and maintenance of the intestinal homeostasis (Thaiss et al., 2016), assuring (i) the mechanical and physiological barrier against colonization with pathogenic agents and overgrowth of existing microorganisms and (ii) anti-tumoral effects, mucosa protection from the side effects and potentiation of antitumoral treatments (Cheng et al., 2020). It is well-known that many factors, from ethnicity, diet, and other lifestyle factors and long-term or frequent use of common antibiotics influence the microbiota composition and can induce malignant transformation (Chelariu et al., 2016, 2017; Sommer et al., 2017; Lazar et al., 2018, 2019; Pircalabioru et al., 2019; Mihai et al., 2021). There are studies stating that the elimination of the infective process with amoxicillin and clarithromycin in early stage of neoplasia has beneficial effects (Van Nuffel et al., 2015). On the contrary, an analysis of 125,441 cancer patients and 490,510 subjects in the control group proved that the antibiotic treatment increased the cancer risk. It has been shown that the incidence of cancer in people treated on long-term with antibiotics increased by 18%, especially for lung, kidney, pancreatic, lymphoma, and myeloma cancers (Friedman et al., 2009). In case of colorectal cancer (CRC), the highest risk was associated with anti-anaerobic antibiotics (vancomycin and penicillins) and the lowest, with tetracyclines (Bufill, 1990; Kilkkinen et al., 2008; Friedman et al., 2009; Dik et al., 2016; Cao et al., 2018).

Neoplasia-associated changes in microbiota are frequently seen, particularly in tissues/systems in contact with gut or respiratory tract microbiota (Wotherspoon et al., 1991; Bayerdörffer et al., 1995; Isaacson and Du, 2004; Yamamoto and Schiestl, 2014; Mima et al., 2017; Mao et al., 2018). The decrease of mucopolysaccharide layer thickness exposes epithelium to direct contact with microbiota and to the development of invasive biofilms (Costerton et al., 1999). It has been shown that the adenomatous polyps, CRC, and healthy epithelium at distance from benign or malignant tumors are covered with biofilms formed by tumorigenic invasive polymicrobial associations incorporated in a polysaccharide matrix (Bufill, 1990; Swidsinski et al., 2005; Dejea et al., 2014, 2018; Tomkovich et al., 2019). In the colon epithelium covered with invasive biofilms, the density of E-cadherin adherence molecules decreases, and the level of IL-6, a marker of increased permeability and inflammatory reaction, increases. By increasing the permeability of the epithelium, the excess lipopolysaccharides (LPS) from the outer membrane of the Gram-negative bacterial cell pass into the blood and induce endotoxemia, inflammation in visceral adipose tissue, disruption of glucose metabolism, insulin resistance, and obesity, playing a role in the development of metabolic syndrome, type 2 diabetes, inflammatory bowel disease, autoimmunity, and carcinogenesis (Halmos and Suba, 2016). The risk of such pathologies is increased by prolonged antibiotic exposure (Hernandez, 2016).

Dysbiosis associated with an increase in pathogenic bacteria such as *Fusobacterium nucleatum* stimulates the growth of different intestinal tumor types, including CRC, by inhibiting the activity of NK cells.

During dysbiosis, the pro-inflammatory Th17 and anti-inflammatory Treg lymphocytes ratio changes in the favor of the last ones, probably due to changes in the concentration and ratios of short-chain fatty acids (SCFAs). It has been shown that butyrate and propionate, but not acetate, stimulate extrathymic production of foxp3 anti-inflammatory Treg lymphocytes (Arpaia et al., 2013). Importantly, SCFA can act as histone deacetylases inhibitors to induce hyperacetylation of histones modulating gene expression leading to apoptosis and growth arrest (Schilderink et al., 2013).

### Modulation of gut microbiota by antitumoral agents and influence of antibiotics

Antitumoral treatments influence the eubiosis by different mechanisms such as: (a) alteration of the quantitative and qualitative panel of molecules produced by the epithelium; (b) induction of changes in gut microbiota, followed by increase of the epithelial barrier permeability, facilitating bacterial translocation into the internal environment in cancer patients that are already immunosuppressed due to cytostatic treatment or malignancy, hence increasing the risk of sepsis (Sullivan et al., 2001; Samet et al., 2013; Papanicolas et al., 2018); (c) alteration of immunological homeostasis due to antibiotic treatment, increasing immunosuppression and susceptibility to infectious diseases and neoplasia (Willing et al., 2011; Alexander et al., 2017); (d) decrease in the efficacy of some anticancer agents and an increase in their toxicity; and (e) triggering of oxidative stress (ROI release) and genotoxicity since bacterial DNA double-strand breaking under antibiotic/cytostatic agents action activate the mutation-inducing SOS repair system leading to antibiotic resistance, while other antibiotics favor the spread of resistant strains (Podolsky, 2002; Cho, 2008).

Some of the most frequently reported alterations of gut microbiota composition and diversity induced by chemotherapy are (i) increased abundance of pathogenic bacteria (e.g., enterococci, staphylococci, *Escherichia coli*, other Enterobacteriaceae, *Pseudomonas*, clostridia, and Gram-negative anaerobes); (ii) augmented bacterial translocation to mesenteric lymph nodes and spleen, accompanied by the occurrence of Th1 and Th17 immune responses (Panebianco et al., 2018); (iii) decreased gut bacterial total numbers and biodiversity; (iv) decrease of beneficial bacteria, such as *Lactobacillus* spp., *Bacteroides* spp., *Faecalibacterium prausnitzii*, *Bifidobacterium* spp., and Firmicutes (Panebianco et al., 2018). It has been shown that oncologic patients harbor a marked reduction in intestinal bacterial diversity, with >30% of fecal samples dominated by a single bacterial genus, increasing the risk of bacterial translocation and explaining the fact that bloodstream infections are causing death in ~10% of cancer patients (Liang et al., 2019). In 50% of patients, bacteremia was preceded by intestinal domination with a corresponding organism for 7 days, the enterococcal domination of the gut microbiota being associated with a 9-fold increase in the risk of vancomycin-resistant enterococcus (VRE) bacteremia, while proteobacterial domination with a 5-fold increase in the risk of Gram-negative bacteremia (Liang et al., 2019).

The majority of antitumoral agents have been shown to modulate the gut microbiota. Anthracyclines have a bacteriostatic effect on the microbiota. Irinotecan is toxic to commensal microbiota, causing an increase in the number of potentially pathogenic species (Enterobacteriaceae, *Clostridium*, and *Fusobacterium nucleatum*). Cyclophosphamide, an alkylating compound, is located at the intersection of immunotherapy and chemotherapy. Its anti-neoplastic efficacy is based on stimulating the anti-tumor immune response, which is mainly pro-inflammatory. As a consequence, it also alters the permeability of the small intestine and induces translocation of Gram-negative and Gram-positive species into the secondary lymphoid organs, where bacteria stimulate the Th1 and Th2 memory lymphocytes. Germ-free mice and conventional mice treated with anti-Gram-positive antibiotics have minimal Th17 lymphocyte response, and tumors do not respond to cyclophosphamide (Viaud et al., 2013).

Besides their direct effect on gut eubiosis, cystostatic derivatives are eliminated by biliary secretion in the gut and could, on their turn, stimulate or inhibit the microbiota (Alexander et al., 2017).

Many studies demonstrate that antibiotic treatment could influence the efficiency of cytostatic treatments. For example, cyclophosphamide efficiency was diminished by vancomycin and colistin, while *Lactobacillus murinus, Lactobacillus johnsonii, Barnesiella intestinihominis*, and *Enterococcus hirae* increased its antitumor activity or restored the response to cyclophosphamide in tumor-bearing antibiotic-treated mice (White et al., 2008; Viaud et al., 2013; Li et al., 2021). The cytotoxicity of CB 1954 was strongly enhanced by *E. coli* nitroreductase activity (Chen et al., 2004). Therefore, gut microbiota modulation could maximize the response to anticancer treatments. For example, starting from the capacity of intravenously injected spores of strictly anaerobic *Clostridium* species to germinate in the hypoxic regions present in solid tumors, a genetically engineered, non-toxinogenic strain of *C. novyi-NT* has been used to carry the anticancer drug and deliver it inside the tumor (Staedtke et al., 2016; Feng et al., 2021). Not only chemotherapy, but also radiotherapy has been shown to induce persistent selective killing of commensal anaerobes as well as expansion of potentially pathogenic enterococci and Enterobacteriaceae (Garajova et al., 2021).

### Influence of gut microbiota on antitumoral treatment efficacy

It has been shown that gut microbiota influences the efficacy of conventional chemotherapy, immunotherapy, radiotherapy and surgery, the drug toxicity, and utltimately, the oncologic patient’ prognosis. Gut microbiota influences the efficacy of chemotherapy and immunotherapy due to its ability to metabolize drugs and xenobiotics, and to modulate host inflammation and immune responses.

The healthy microbiota enhances the efficacy of the platinum-based agent oxaliplatin, by inducing ROI release from myeloid cells, thereby enhancing inflammatory cytokine production and tumor regression (Goldszmid et al., 2015). This effect was decreased in germ-free / antibiotic-treated mice, while a better response of mice to cisplatin was obtained when combined with *Lactobacillus* (Iida et al., 2013). On the contrary, *Fusobacterium nucleatum* has been shown to increase resistance platinum-based agents through increasing authophagy (Yu et al., 2017).

Doxorubicin has been shown to be inactivated by microbial deglycosylation performed by strains of *Streptomyces* and *Raoultella* (Westman et al., 2012; Blaustein et al., 2021).

The efficacy of 5-Fluorouracil has been reduced by the presence of mycoplasmas or *F. nucleatum*, *via* upregulation of an inhibitor apoptotic protein (IAP). It has been shown that *F. nucleatum* increases tumor-associated neutrophils, dendritic cells, and pro-cancer M2 macrophages and prevents the cytotoxicity of T and NK cells (Zhang et al., 2019). When present inside the tumor microenvironment, *Mycoplasma hyorhinis* can induce a dramatic antitumor activity decrease (20–150-fold) of 5-Fluorouracil by degradation to its less active derivatives (Bronckaers et al., 2008; Garajova et al., 2021).

Gemcitabine can be metabolized by different microbes, such as *Mycoplasma, F. nucleatum*, and *E. coli* which express nucleoside analog-catabolizing enzymes to its inactive metabolite 2′,2′-difluoro-2′-deoxyuridine (dFdU) and resistance could be neutralized by some antibiotics such as ciprofloxacin, which eliminate these species from gut microbiota (Geller et al., 2017; Garajova et al., 2021).

Gut microbiota dysbiosis has been also shown to interfere with the efficacy of anticancer immunotherapy (Garajova et al., 2021), inducing resistance to antibodies against programmed cell death 1 (PD-1; Routy et al., 2018). Moreover, the efficacy of Ipilimumab, a monoclonal antibody that neutralizes the cytotoxic T-lymphocyte-associated protein 4 (CTLA-4), is dependent on the presence of *Bacteroides fragilis* polysaccharide that induces a splenic Th1 cell memory response (Beck et al., 2006; Vetizou et al., 2015). Besides increasing its efficiency, many members of gut microbiota such as *Bacteroidaceae*, *Rikenellaceae*, and *Barnesiellaceae* protect from Ipilimumab adverse events (Dubin et al., 2016). The favorable clinical outcome during anti-PD1 therapy was linked to the presence of *Akkermansia muciniphila*, as well as to high diversity and abundance of *Clostridiales/Ruminococcaceae*/*Faecalibacterium, Bifidobacterium longum*, *Collinsella aerofaciens*, and *Enterococcus faecium*. Conversely, the poor responders harbored a low diversity and high abundance of *Bacteroidales* (Robert et al., 2011; Sivan et al., 2015; Gopalakrishnan et al., 2018; Mao et al., 2021; Wong et al., 2021; Sevcikova et al., 2022).

### Chemotherapy and emergence of AMR

Chemotherapy could trigger the emergence of *de novo* antimicrobial resistance, one of the mechanisms being the increase of mutation rate by activating the bacterial SOS response to DNA damage (Poulin-Laprade et al., 2015). Mitomycin C has been shown to be a potent inducer of the bacterial SOS response, being used to select resistant *E. coli* clones with resistance mediated by mdfA efflux pump overexpression (Wei et al., 2001). It has been shown that activation of the SOS response by mitomycin C increases the transcription of genes necessary to horizontal transfer the SXT element that encodes resistance to multiple antibiotics in *Vibrio cholerae* (Beaber et al., 2004). Methotrexate, a dihydrofolate reductase inhibitor, acts by blocking dihydrofolic acid synthesis similarly to trimethoprim antibiotic and thus, co-selects bacterial cells carrying the trimethoprim resistance gene on the same plasmid (Guethmundsdottir et al., 2021). Non-lethal doses of cisplatin resulted in a 3-7-fold increase in mutation frequency, leading to resistance to rifampicin and ciprofloxacin and the administration of antioxidants (ascorbic acid) decreased genotoxicity by 41% and bacterial mutation rates by 65% (Lofmark et al., 2006; Chistyakov et al., 2018). Some chemotherapeutic agents have been shown to increase the horizontal transfer of bacterial resistance genes by phage transduction, transformation with external DNA, or plasmid transfer by conjugation (Wei et al., 2001).

## Conclusion

The evolutionary strategies used by bacteria and tumors to individually and collectively adapt to continuously changing and stressful environments share many similarities, opening new avenues in the study of drug resistance within cancer tissues, using more simple and reproducible bacterial models, as well as in the development of novel antitumor agents and microbiome-based therapeutic interventions that may be able to correct dysbiosis and thus to maximize the treatment efficiency and prevent selection of drug resistance. The gut microbiota is influenced by and influences the treatment efficacy and drug toxicity. Chemotherapy is likely to produce *de novo* antimicrobial resistance in gut microbiota by inducing dysbiosis, increasing the horizontal gene transfer and the mutation rates consequently to the bacterial SOS system activation. On the other side, the disruption of commensal gut microbiota and alteration of host physiology might influence both the efficacy of the antitumoral treatments and their toxicity. Therefore, a better knowledge of the complex interactions among gut microbiota, antibiotics, and anticancer drugs will enable us to develop novel anticancer treatment strategies and subsequently improve the cancer patients’ outcome, minimizing the risk of antibiotic-resistant bacteria carriage and of associated infections.

## Author contributions

MCh, GM, NC, and RF original draft preparation. EI, LB, and CB reviewing and editing. GP figures and manuscript submission. MCo figure preparation. All authors contributed to the article and approved the submitted version.

## Funding

This research was funded by CNFIS-FDI-2022-0675, UEFISCDI - PN-III-P4-PCE2021-1797, PN-III-P1-1.1-36PD-2019-0499, Grant number 224/2021 and the Ministry of Research, Innovation, and Digitalization through Program 1—Development of the national R&D system, Subprogram 1.2—Institutional performance—Financing projects for excellence in RDI, Contract no. 41 PFE/30.12.2021. The funders had no role in the design of the study; in the collection, analyses, or interpretation of data; in the writing of the manuscript; or in the decision to publish the results.

## Conflict of interest

The authors declare that the research was conducted in the absence of any commercial or financial relationships that could be construed as a potential conflict of interest.

## Publisher’s note

All claims expressed in this article are solely those of the authors and do not necessarily represent those of their affiliated organizations, or those of the publisher, the editors and the reviewers. Any product that may be evaluated in this article, or claim that may be made by its manufacturer, is not guaranteed or endorsed by the publisher.

## Footnotes

^1^ https://www.who.int/news-room/fact-sheets/detail/cancer

^2^ https://www.who.int/news-room/fact-sheets/detail/antimicrobial-resistance

^3^ https://www.reactgroup.org/news-and-views/news-and-opinions/year-2020/new-react-policy-brief-successful-cancer-treatment-relies-on-effective-antibiotics/
